# MDCT of Small Bowel Obstruction: How Reliable Are Oblique Reformatted Images in Localizing Point of Transition?

**DOI:** 10.1155/2014/815802

**Published:** 2014-05-06

**Authors:** Wasim Memon, Yasir Jamil Khattak, Tariq Alam, Luca Maria Sconfienza, Muhammad Awais, Shayan Sirat Maheen Anwar

**Affiliations:** ^1^Aga khan University, Karachi, Pakistan; ^2^Division Servizio di Radiologia, IRCCS Policlinico San Donato, Organization Università degli Studi di Milano, Milano, Italy

## Abstract

The goal of this study is to prospectively assess the additional value of oblique reformatted images for localizing POT, having surgery as a reference standard. *Materials and Methods*. 102 consecutive patients with suspected small bowel obstruction (SBO) underwent 64-slice multidetector row CT (MDCT) using surgical findings as reference standard. Two independent GI radiologists reviewed the CT scans to localize the exact POT by evaluating axial images (data set A) followed by axial, coronal, and oblique MPR images. CT findings were compared to surgical findings in terms of diagnostic performance. McNemar's test was used to detect any statistical difference in POT evaluation between datasets A and B. Kappa statistics were applied for measuring agreement between two readers. *Results*. There was a diagnostic improvement of 9.9% in the case of the less experienced radiologist in localizing POT by using oblique reformatted images. The more experienced radiologist showed diagnostic improvement by 12.9%.

## 1. Introduction

Small bowel obstruction (SBO) is a common clinical condition as a cause of abdominal pain, accounting for approximately 20% of all emergency admissions for acute abdomen. It is also amongst one of the commonest bowel pathologies leading to surgical consultation [1–3].

Early diagnosis of SBO obstruction is essential to prevent bowel ischemia. In the past, the paradigm was to “never let the sun set or rise on an obstructed bowel.” This probably was an evidence of limitations regarding availability of various imaging modalities for diagnosing the exact site of SBO [4].

Although plain X-ray abdominal evaluation still remains the investigation of first choice in cases of suspected SBO due to its low cost and wide availability, it cannot reliably diagnose the exact level of obstruction and thus can only serve as a basis for triage for further imaging workup [5, 6].

With the ongoing developments in imaging techniques overtime, computed tomography (CT) has emerged as an excellent modality in the diagnosis of SBO. CT scan not only reliably diagnoses SBO but can also be of great help in determining the cause, severity, and the precise point of obstruction [7–10]. Localizing the point of transition (POT) is empirical as it increases confidence in diagnosis, guides patient care, and thus helps in further management.

Ongoing dilation of the intestine increases luminal pressures. When luminal pressures exceed venous pressures, loss of venous drainage causes increasing edema and hyperemia of the bowel. This may eventually lead to compromised arterial flow to the bowel, causing ischemia, necrosis, and perforation. One such example is closed-loop obstruction, in which a section of bowel is obstructed proximally and distally. In such cases there may be few presenting symptoms with rapid progression to ischemia. Localizing the point of transition is imperative to pick the diagnosis in these patients.

Another important reason for finding the discrete transition point is that it helps guide operative planning. The number of points of transition being either one or more is also helpful in deciding between laparotomy and laparoscopic surgery.

In certain cases of extensively dilated bowel loops or lean and thin patients in whom interfaces between bowel loops are very thin due to paucity of intraperitoneal adipose tissue, it may be challenging to localize the POT reliably using axial slices alone [11, 12].

With the currently available multidetector CT (MDCT) scanners, we can get near-isotropic voxels, which can be less than a millimeter in dimension and thus can produce multiplanar reformations with spatial resolution similar to axial sections. This may potentially enhance the role of reformatted images in localizing POT.

Thus, the purpose of this study was to prospectively assess the additional value of oblique reformations from isotropic voxels obtained using a 64-slice MDCT to localize POT, having surgery as a reference standard [13–16].

## 2. Materials and Methods

This was a cross-sectional study carried out between January, 2008, and July, 2011, at Aga Khan University Hospital, Karachi. All patients who entered the emergency department with strong suspicion of SBO on plain radiographs and underwent a CT examination for evaluation of SBO were included in the study. A total of 187 consecutive patients were considered for inclusion in our work. Among these 187 patients, 102 underwent surgery. The remaining 85 patients were conservatively managed and were therefore excluded. CT examinations were performed using a 64-slice MDCT (Toshiba Aquilion 64) without oral contrast administration unless specifically requested by the surgical team. Images were acquired starting from the diaphragmatic dome extending to the pubic symphysis with section thickness of 5 mm at 5 mm interval with beam pitch of 1.5, rotation time of 5 seconds using 120 kV, and 350 mA and 175 mAs and WL = 340/40. All patients received 1.5–2 mL/kg body weight of nonionic contrast (name, brand) warmed to body temperature, injected at a rate of 3-4 mL/s using a mechanical power injector (name, brand) through a 20 G cannula inserted into an antecubital vein. Images were acquired in arterial and portovenous phases using 10-second delay for arterial and 65 seconds for delayed phases. After the raw data was acquired, reconstructions were performed.

The axial sections, that is, the raw data, were reconstructed in two steps: first with 5 mm-thick sections at 5 mm intervals in the transverse plane followed by 0.5 mm thick sections at 1.5 mm intervals.

This second dataset of reconstructed axial sections scans was then used to acquire coronal reformations in the coronal plane with a thickness of 3 mm at 1.5 mm intervals using soft tissue algorithm. The acquisition of reformations is part of the routine MDCT protocol in our department. All these reconstructions were performed by the technologist at the CT console with a commercially available console system devoted to rapid reconstruction and later sent to picture archive and communication system (PACS). Oblique reformations were performed by the readers at the Vitrea station from available data. We were able to get near-isotropic voxels, producing multiplanar reformations with spatial resolution similar to axial sections. This potentially enhanced the role of reformatted images in localizing POT. Two independent radiologists with 10 and 12 years of experience in abdominal imaging reviewed the images. Both observers were blinded to surgical findings and reviewed two different datasets (A, including axial and coronal images only; B, volume data for oblique reconstructions in addition to dataset A) with an 8-week interval to prevent recall bias. Reviewing radiologists were blinded to surgical findings of the level of obstruction. CT was evaluated to localize the exact location of the point of transition (POT) and etiology of obstruction. Also, readers were asked to rate the confidence of localizing and reporting the POT after use of data set B using a semiquantitative scale (increased, not changed, and decreased). CT findings were compared to surgical findings in terms of diagnostic performance. Interpretations were compared with surgical findings by primary researcher to evaluate for accuracy. McNemar's test was used to detect any statistical difference in POT evaluation between datasets A and B. Kappa statistics were applied for measuring agreement between two readers for their findings of localizing the POT. SPSS version 11 was used for statistical analysis. A *P* value <0.05 was considered significant.

## 3. Results

The commonest cause of SBO in the study group was adhesions found on laparotomy (*n* = 59), followed by hernias (*n* = 17) and small bowel obstruction secondary to tuberculosis (*n* = 16). Other causes included postradiation stricture formation, gall stone ileus, tumor, volvulus, abscess formation, and foreign body/bezoars (Table 1).

### 3.1. Cause of Small Bowel Obstruction in the Study Group

A total of 102 cases of surgically proven SBO were included in the study. Among these 102 patients, the less experienced radiologist correctly localized the transition zone in 85 cases (84.2%) using data set A and 95 cases when using data set B (94.15%), with a 9.9% diagnostic improvement (95% CI 2.7%−11.6%).

The more experienced radiologist correctly localized the transition zone in 83 cases (82.2%) using data set A, versus 96 cases (95.0%) using data set B, improving by 12.9% with 95% CI of 6.5% to 12.9% (Table 2).

### 3.2. Improvement in and Accuracy of Detecting Point of Transition after Using Data Set B

**Figure 1:**
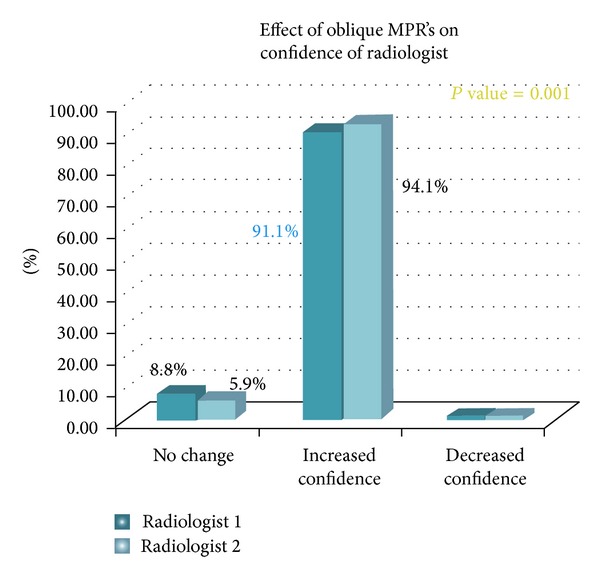
Effect of oblique MPRs on confidence of radiologist in diagnosing point of transition.

**Figure 2:**
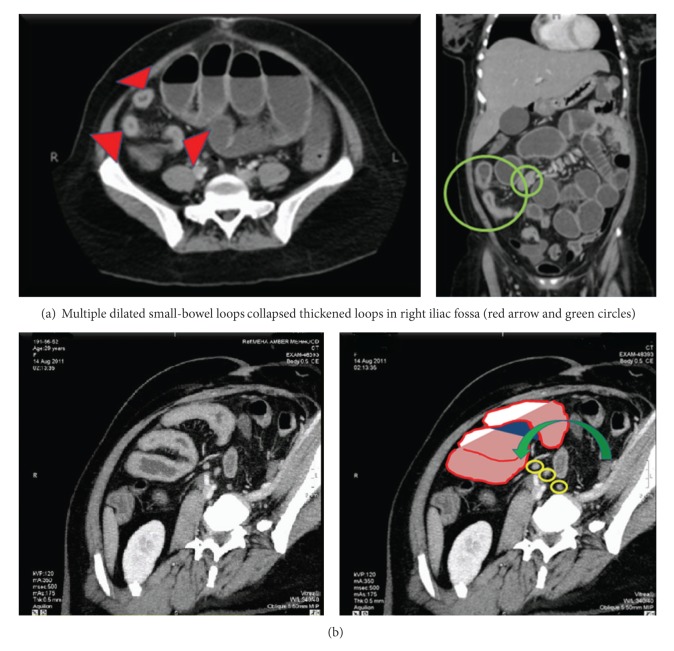
CT scan of 48-year-old female with acute abdominal pain. (a) Axial and coronal CT images obtained with intravenous contrast agents show dilated small-bowel loops (red arrow heads and green circles). (b) Oblique reformation shows dilated small-bowel loops with a transition point (green arrow) in the midabdomen. Multiple small mesenteric lymph nodes also visualized (yellow circles).

**Figure 3:**
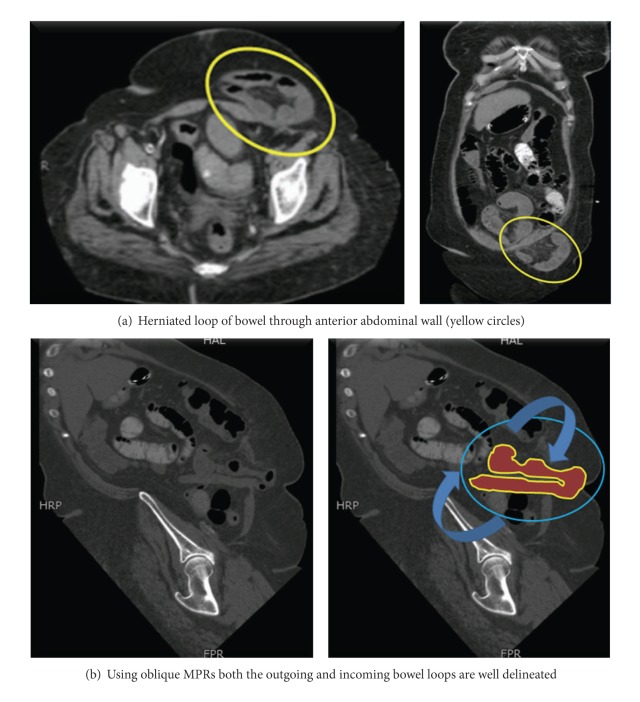
CT scans in a 54-year-old woman with a two-month history of nausea and vomiting acutely presenting with worsening of symptoms. (a) Axial and coronal CT images obtained with intravenous contrast agent show herniated loops of small-bowel loops through a defect in anterior abdominal wall; however, the tract of bowel could not be completely elucidated on axial and coronal sections alone (yellow circles). (b) Oblique reformation exactly shows the outgoing and incoming loops of bowel with proximal dilated segments of bowel representing partial obstruction (blue circles and curved arrows).

When evaluating dataset B, the less experienced radiologist reported increased confidence in diagnosis of POT in 93 cases and similar confidence in 83 cases compared to the evaluation of dataset A. The more experienced radiologist reported increased confidence in diagnosis of POT in 95 cases and similar confidence in 82 cases compared to the evaluation of dataset A. The confidence scores for the presence of point of transition for both radiologists were higher after using data set B (axial, coronal, and oblique reformatted images) as compared to data set A (only axial and coronal images). Thus use of oblique reconstructions enhanced confidence in localizing POT (*P* = 0.001) (Figures 1, 2, and 3).

**Figure 4:**
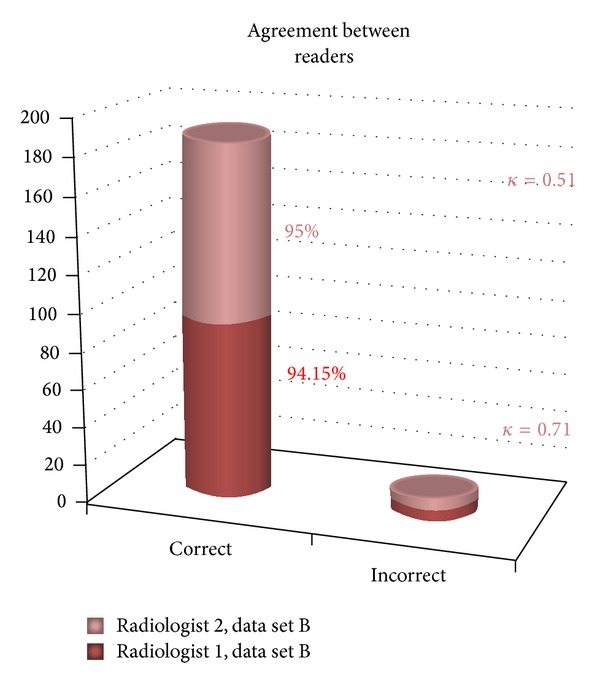
Agreement between readers.

Kappa statistics for the measurement of agreement were found between readers for both sets A and B. For the data set A, there was good agreement between both radiologists (value = 0.51). For the data set B, the value was even higher, 0.71, indicating a higher level of agreement after using MPRs (Figure 4).

## 4. Discussion

Small bowel obstruction is one of the commonest reasons for presentation to the emergency department. Even today with the recent advances in imaging the diagnosis is made on the basis of clinical signs and symptoms, with plain radiography as the initial approach. However regarding the management of SBO, it is imperative not only to diagnose its severity but also to localize the exact site of obstruction which is an important determinant of the prognosis and help to the surgeon [17, 18].

With CT it has now become possible to not only reliably diagnose SBO but also acquire multiplanar reformations. Technological advances have crept up the ladder with time, making us available scanners like 64-slice MDCT and above. This paradigm shift has negotiated the problems like limited *z*-axis resolution, longer acquisition times allowing submillimeter isotropic data which not only is of great diagnostic help in cardiac, pulmonary, and musculoskeletal pathologies by facilitating better evaluation of anatomy but also has applications with respect to the abdomen and pelvis like pancreatic and gastric cancers as described by Prokesch et al. and Kim et al. in their respective studies [19–22].

Coronal reformatted images have also proved to be helpful in increasing confidence level of readers for the diagnosis of acute appendicitis [23].

In the literature several studies have evaluated the role of multiplanar reconstructions in SBO. Lazarus et al., Caoili and Paulson, and Furukawa et al. in their studies suggested that multiplanar reformations are helpful in the localization of the transitional zone. Jaffe et al. proved isotropic coronal multiplanar reformatted images to have additional diagnostic value in cases of bowel obstruction showing better agreement among independent observers especially for the diagnosis of level and cause. A recent study by Hodel et al. specifically evaluated the role of CT reformatted images in small bowel obstruction using 16-slice MDCT and reported an increase in both accuracy and confidence in the localization of the transition zone in CT of mechanical SBO [11, 13, 24–26].

Compared to the study by Hodel et al., the cases included in this study were all surgically proven with perioperative findings taken as the gold standard for point of transition. Moreover the study was performed with a 64-slice MDCT as opposed to a 16-slice MDCT.

Although some radiologists and surgeons would consider coronal sections to be a better view regarding display of bowel since Coronal view is almost analogous to the frontal view of an abdominal radiograph and is synonymous to the plane learned in surgical training, Coronal view cannot be employed alone in cases of SBO since all the parts of bowel are not included on an isolated reformatted view. This could lead to confusion of narrowed part with an adjacent structure [27].

We thus included both the axial and coronal images in data set A, to make both data sets as similar as possible. This would also help to more correctly elucidate the additional value of oblique MPRs. Sagittal sections were however excluded from the data sets since they do not display more bowel loops in a single image and additionally it is difficult to localize the bowel loop anatomically as compared to axial and coronal images making it more time-consuming with little potential improvement in diagnosis.

Since the study went on for duration of more than 4 years, image grid overload with volume data was one of the prime challenges the researchers were confronted with. To deal with this all the available volume data was saved in compact discs while removing it from the image grid every 3 months and then reloading it onto the workstation for making oblique reconstructions at the time of recording the findings. To reduce the image load the axial data was presented on the PACS as 5 mm-thick sections. Reconstructions were performed from the available source images which were 1.5 mm thick.

We had in the study group myriad causes of SBO proven either by surgery or histopathology, amongst which adhesions were the commonest cause followed by abdominal hernias and tuberculosis. Although SBO was studied in different data sets, the radiologist was not asked to record the probable cause of obstruction on available images since it was not the prime objective for the study.

We were able to achieve significantly improved accuracy in localization of transitional zone following use of MPRs by both radiologists with good agreement between the two. Although there was a difference between the two radiologists regarding experience, in our study no significant difference was found with respect to the results for both data sets A and B. We were also able to find that MPR enhances radiologist's confidence in calling a particular level point of obstruction which corroborates results given in studies by Jaffe et al., Paulson et al., and Hodel et al.

All the 102 patients included in our study underwent surgery, amongst which 19 patients had multiple levels of obstruction mostly due to adhesions. It was difficult to pick all the transition in cases where there was more than a single point of obstruction. This factor together with cases with extensive dilatation of bowel loops (high grade obstruction) could have been the reason for reduced CT accuracy in the first data set (84.1% and 82.1%) [3, 7, 14, 28].

One of the limitations of our study is that we did not classify the bowel obstruction into its grades of severity. By classifying the obstruction into various grades we could correlate the accuracy rates for various grades using MPRs as well.

## 5. Conclusion

In conclusion it was found that although the oblique MPR increases the image load and is time-consuming it surely is a development and a new perspective of utilizing the available information whereby it can significantly prove as a powerful adjunct in diagnosis and management of SBO.

## Figures and Tables

**Table 1:** Cause of small bowel obstruction in the study group.

Cause	Frequency	Percent	Valid percent
Adhesions	59	57.8	57.8
Hernia	17	16.7	16.7
Volvulus	1	1.0	1.0
Tuberculosis	16	15.7	15.7
Tumour	2	2.0	2.0
Abscess formation	1	1.0	1.0
Foreign body or bezoars	1	1.0	1.0
Post radiation	3	2.9	2.9
Gall stone ileus	2	2.0	2.0

Total	102	100.0	100.0

**Table 2:** Improvement in accuracy of detecting point of transition after using data set B.

Improvement in accuracy					
Readers	Data set A	Data set B	Improvement	McNemar values	Cl values
Experienced radiologist	82.2%	95%	12.9%	0.002	6.5%–12.9%
Less experienced radiologist	84.2%	94.15%	9.9%	0.006	2.7%–11.6%
